# CRISPR/*cas* Loci of Type II *Propionibacterium acnes* Confer Immunity against Acquisition of Mobile Elements Present in Type I *P. acnes*

**DOI:** 10.1371/journal.pone.0034171

**Published:** 2012-03-30

**Authors:** Holger Brüggemann, Hans B. Lomholt, Hervé Tettelin, Mogens Kilian

**Affiliations:** 1 Department of Biomedicine, Aarhus University, Aarhus, Denmark; 2 Institute for Genome Sciences and Department of Microbiology and Immunology, University of Maryland School of Medicine, Baltimore, Maryland, United States of America; Charité-University Medicine Berlin, Germany

## Abstract

*Propionibacterium acnes* is a skin commensal that occasionally acts as an opportunistic pathogen. The population structure of this species shows three main lineages (I–III). While type I strains are mainly associated with sebaceous follicles of human skin and inflammatory acne, types II and III strains are more often associated with deep tissue infections. We investigated the occurrence and distribution of the clustered regularly interspaced short palindromic repeats (CRISPR) in *P. acnes*, assessed their immunological memory, and addressed the question if such a system could account for type-specific properties of the species. A collection of 108 clinical isolates covering all known phylotypes of *P. acnes* was screened for the existence of CRISPR/*cas* loci. We found that CRISPR loci are restricted to type II *P. acnes* strains. Sequence analyses of the CRISPR spacers revealed that the system confers immunity to *P. acnes*-specific phages and to two mobile genetic elements. These elements are found almost exclusively in type I *P. acnes* strains. Genome sequencing of a type I *P. acnes* isolate revealed that one element, 54 kb in size, encodes a putative secretion/tight adherence (TAD) system. Thus, CRISPR/*cas* loci in *P. acnes* recorded the exposure of type II strains to mobile genetic elements of type I strains. The CRISPR/*cas* locus is deleted in type I strains, which conceivably accounts for their ability to horizontally acquire fitness or virulence traits and might indicate that type I strains constitute a younger subpopulation of *P. acnes*.

## Introduction

The Gram-positive bacterium *Propionibacterium acnes* is one of the predominant members of the commensal skin microbiota [1], [2]. It successfully colonizes sebaceous follicles of healthy human skin, but is also associated with the formation and/or progression of acne vulgaris and with a number of opportunistic infections [3]–[5]. The apparent contradiction between the pathogenic nature and the role as a ubiquitous skin commensal may be partly explained by strain-specific properties. *P. acnes* strains were categorized as phylotypes I, II and III according to sequence comparison of their *tly* and *recA* genes [6], [7]. More recently, a multilocus sequence typing (MLST) approach, designated the Aarhus scheme, has been used to further discriminate strains, resulting in the identification of clonal complexes (CC) and 57 sequence types (ST) among 210 strains analyzed [8]. Again, three divisions were identified (I, II and III); division I was further subdivided into I-1a, I-1b and I-2. Subdivision I-1a, including the epidemic clone ST 18 and its descendants (CC 18), comprised significantly more isolates associated with moderate to severe acne. In contrast, other phylotypes of *P. acnes* are associated with healthy skin and with opportunistic deep tissue infections, in particular type II strains [8]. These findings were recently confirmed by an independent study, which employed an alternative MLST scheme [9]. A comparison of the two MLST schemes revealed a different discriminatory power of the two schemes and underlined the previous observation that the clonal complexes CC 3, CC 18 and CC 31 are associated with acne whereas CC 36, CC 60 and ST 27 are associated with healthy skin, but also with opportunistic infections associated with medical implant devices [10].

*P. acnes* genome comparison studies revealed the existence of genomic regions with island-like features, indicating that they were horizontally acquired [11], [12]. These islands, encoding putative fitness and pathogenic traits, are only present in a subset of *P. acnes* strains, i.e. in certain CCs. It is unclear so far if these islands are still mobile and could disseminate via horizontal gene transfer (HGT). One powerful system, which restricts the acquisition of incoming DNA, thus of HGT, is a recently discovered ‘adaptive immune’ system, composed of clustered regularly interspaced short palindromic repeats (CRISPRs) and CRISPR-associated genes (*cas*) [13]–[15]. CRISPR/Cas systems are found in many bacteria and archaea, conferring immunity to invasion of a variety of foreign mobile elements, i.e. viruses or plasmids. Whether CRISPR loci exist in *P. acnes* has not been explored so far. Here, we show the exclusive presence of CRISPR loci in type II *P. acnes*, and reveal their immunological memory by analyzing the spacer sequences. Several spacers originated from mobile genetic elements present in a subset of type I *P. acnes* strains. Gene content analysis of these elements points to their potential role as virulence and/or fitness traits.

## Results and Discussion

### Identification of CRISPR/*cas* in *P. acnes* genomes

**Figure 1:**
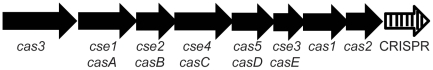
The CRISPR/*cas* gene locus in *P. acnes* J139. Shown is the gene order in strain J139 (locus tags: HMPREF9206_0746 (*cas3*) to HMPREF9206_0752 (*cas1*); *cas2* has not been correctly annotated in the draft genome [GenBank: ADFS00000000]). The gene cluster is identical in all analyzed *P. acnes* genomes. The CRISPR loci are composed of 1–9 repeats and 1–8 spacers.

**Figure 2:**
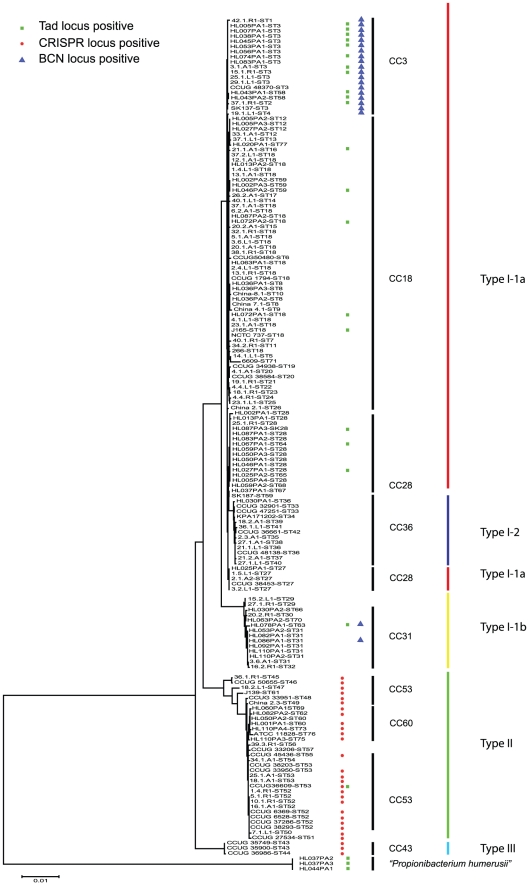
Phylogenetic tree of *P. acnes* strains used in this study and distribution of mobile genetic elements. The tree was constructed based on 4,287-bp concatamer of partial sequences of nine housekeeping genes, and sequences types (STs) were assigned employing the Aarhus MLST scheme (http://pacnes.mlst.net/). The tree was constructed using the Minimum Evolution algorithm in MEGA version 5.05. The bootstrap values were based on 500 replicates and only values exceeding 50 are shown. Clonal complexes (CCs) were determined according to an eBURST analysis described in Kilian et al. [10]. The distribution of three genomic regions within this strain collection was checked by PCR, namely the CRISPR locus (red circle), the TAD locus (green square), and the bacteriocin (bcn) island (blue triangle).

We searched the available genome sequences for the existence of CRISPR/*cas* loci. The completely sequenced genomes of *P. acnes* KPA171202 (KPA), 266, SK137 and 6609 did not contain a *cas* gene cluster, nor CRISPR loci. However, the recently decoded genome of *P. acnes* strain ATCC 11828 [16] as well as some partially sequenced genomes, e.g. strain J139 (reference genome for the Human Microbiome Project (HMP)) contained *cas* loci, composed of the *cas* genes *cas3-casA-casB-casC-casD-casE-cas1-cas2* (Figure 1). The gene cluster composition is identical to the CRISPR/*cas* gene cluster of *E. coli* K12 W3110 [17]. According to the classification of Makarova et al. [18] the system of *P. acnes* belongs to type I, since it encodes Cas3, a protein with separate helicase and DNase activities [19]; it can be further classified as a member of subtype I-E. Downstream of *cas2*, no CRISPR region could be identified in strain ATCC 11828; however, a CRISPR region was identified in strain J139, composed of three identical repeats (GTATTCCCCGCCTATGCGGGGGTGAGCCC) and two spacers. Also other partially sequenced *P. acnes* genomes, which are sequenced in the framework of the HMP (strains HL001PA1, HL060PA1, HL082PA2, HL110PA3, and HL110PA4) contained CRISPR loci with the same repeat in different copy numbers (Figure 2).

### PCR detection of CRISPR loci in clinical isolates of *P. acnes*

Since we had no information on the origin and clinical relevance of the five CRISPR/*cas*-positive *P. acnes* strains of the HMP, we decided to PCR-screen our strain collection of 108 clinical *P. acnes* isolates for the existence of CRISPR regions. The strain collection contained clinical isolates from diverse tissue and disease sites, e.g. healthy skin, acne vulgaris, foreign body/joint infections, wound infections, endocarditis, oliocranian bursitis, urinary tract infection, and meningitis (Table S1). All isolates were previously typed according to Aarhus MLST scheme [8], [10]; all known phylo-/sequence types of *P. acnes* were represented in the strain collection as illustrated by a phylogenetic tree based on concatamers of sequences of nine housekeeping genes (Figure 2). The two primers for CRISPR amplification were derived from sequences up- and downstream of the CRISPR region in strain J139; it was ascertained that the primer sequences are conserved in the CRISPR/*cas*-positive *P. acnes* genomes of the HMP.

We obtained PCR products for almost all strains of the sequence types ST 43 to ST 55, representing type III (STs 43, 44) and type II strains (STs 45–53 and ST 55) (Figure 2, Table S1). No PCR product was obtained with any type I strain, nor with type II strains of STs 54, 56, and 57. The five partially sequenced CRISPR/*cas*-positive genomes of the HMP are also exclusively type II strains (Figure 2), thus supporting our conclusion, that the CRISPR/*cas* locus is restricted to type II/type III *P. acnes*.

### Sequence analysis of CRISPR spacer sequences

Subsequently, all PCR products were sequenced. All sequences contained one to nine copies of the repeat sequence GTATTCCCCGCCTATGCGGGGGTGAGCCC. Type III strains (STs 43, 44) contained no spacer, whereas the type II strains had one to maximal eight spacer sequences (Table S2). The standard spacer length was 32 bp. Altogether, 63 spacers in 19 type II strains were identified. A total of 25 distinct spacer sequences could be identified (Table 1) and several spacers detected in different strains were identical; for example spacer no. 4 ((G)AGGGCTACCACGTGGTCGATTTGGACTGTCG) was present in seven different strains (Table S2). All distinct spacer sequences were blasted to nucleotide databases: nine spacers had no hit in any database, four of them matched to *P. acnes* phages, three spacer sequences matched to mobile elements of other bacteria, one matched to an island-like region in the genome of SK137 (type I, ST 3) and eight matched to a special genomic region in several type I *P. acnes* genomes. All categories are discussed below.

**Table 1:** CRISPR spacers identified in type II *P. acnes* strains.

Strain	ST	source	Spacer sequence	#	BLAST result	Comment*
36.1.R1	45	Acne mild	CGGCCTGCGGCAGATTTTTGTTGCGTTGAATCC	1	phages PAS50, PAD20, PA6	
			CGGGCAGAGGATGTGTTGCTCGTTCCTGGATGG	2	phages PAS50, PAD20, PA6	
			GTTACGCTGGAACCCCCAATGAACACGCGAGAA	3	phages PAD20, PAD42, PAS40,etc	
			GAGGGCTACCACGTGGTCGATTTGGACTGTCG	4	*P. acnes* SK137	bacteriocin locus
			CAGGCGCTCCACTCCCTCGCCCTGGCCACCAAC	5	No hit	
18.2.L1	47	Healthy skin	ACCGGGCCCATCCCGGTCGGCCTCCTGAAAGG	6	type I *P. acnes* genomes	TAD locus
			TGGCTAGTACGGCCACGGATGAGATTGAGGCC	7	type I *P. acnes* genomes	TAD locus
			GTGAACGGGGCATGGGATTAGCCGAGGCGCTA	8 (2×)	type I *P. acnes* genomes	TAD locus
			ACCACTCGGGGTGGGACTGCCCAGTTTTATTG	9 (2×)	No hit	
			GCCTACCGTCAGCTGACTCACGCCTCCGCGTT	10	type I *P. acnes* genomes	TAD locus
			TCACACCAGTCATCAGCGTCATAGTCCTCTCGG	11	No hit	
CCUG33951	48	Blood	CCATGAGCGGCTGCGCTCCCGATCGGCGGCG	12	type I *P. acnes* genomes	TAD locus; (5)
10.1.R1	52	Healthy skin	TTGGGTGGGTGAGGTCGGGTCGTCAGTCATGAG	13	*Verrucosispora maris* AB-18-032	*cse3*
			ACGTCGTGAACGTACCCCTTGACGGAGACGGCA	14	No hit	
			CGGTGTTAACGGCTTGCCTGGCTTGGATGGAGC	15	No hit	
			CCCATACTGTGCGGGTTGGCGACTATCTGTGGA	16	No hit	
1.4.R1	52	Healthy skin	GTCGATGTCGAGATTGGCCTGGGGGTCCATGTC	17	type I *P. acnes* genomes	TAD locus; (13, 14)
CCUG6528	52	Acne	CCAGACAACCTCGACAACCTGTTCAGGGGATG	18	phage PAS50	(2, 3, 4, 5)
			ATGGCTAGCCCGGATTTTTGGCTGCCTGAGCG	19	*Porphyra haitanensis* PH-41	microsatellite sequences
CCUG37286	52	Blood	GTCGACCAGACCCGGATCGGGCGTTTAGGTCG	20	No hit	(16)
CCUG6369	52	Abscess	ATCTGCCAACGAGCGAGAGTGGCGCGGTGTTC	21	*Acidiphilium multivorum* AIU301	plasmid pACMV1; (4, 5)
25.1.A1	53	Acne mild	CGACTACCTACGGTTGGCCACCGAAATCAGTG	22	type I *P. acnes* genomes	TAD locus
			GCCTCGATCACCGGGCTGGTCGGCGTTCAGGA	23	type I *P. acnes* genomes	TAD locus
			TGCGCTGTAGACATGATCATTCCCCCGCTCTC	24 (3×)	No hit	
			AGCACCTCATCCTGTCCGCCGGCACGCCACCC	25	No hit	

* numbers in brackets indicate additional spacers present in the respective strain (see Table S2 for a complete overview).

### Phage-DNA specific spacers

The CRISPR/Cas system of type II *P. acnes* apparently confers immunity against a variety of mobile, “foreign” DNA elements. One category of foreign DNA is phages: we detected six spacers (four unique sequences, no. 1, 2, 3, 18) in three different strains whose sequence matched with known *P. acnes* phages, such as the sequenced siphoviruses PAD20 and PAS50 [20]. *P. acnes* phages are a very homologous group of viruses, and are distinct from phages isolated from other propionibacteria. It was found that 70% of *P. acnes* strains have inducible siphoviruses, which most likely have a pseudolysogenic life cycle [20], [21]. These phages have a strong lytic activity against all *P. acnes* isolates with inducible phages, but isolates with no prophages are less susceptible. That might indicate that the phage-resistant strains of *P. acnes* contain the CRISPR/Cas system. It has to be determined if resistant strains do indeed possess CRISPR loci with phage-derived spacer sequences.

### Protospacer within a genomic island present only in type I (CC 3/CC 31) *P. acnes* strains

We found one spacer (no. 4) that was present in seven different strains. The spacer sequence matched to an island-like region in the genome of type I strain SK137 (ST 3). This 20.7 kb island was described previously in a genome comparison study [12], and contains a gene cluster for bacteriocin synthesis (Figure S1). The island is inserted into the *P. acnes* core genome, and disrupts a gene (PPA0155 in strain KPA) encoding an ATP-dependent helicase. We blasted this island also against all partially sequenced *P. acnes* genomes and found it in 13 strains (HL078PA1, HL045PA1, HL007PA1, SK182, HL083PA1, HL005PA1, HL074PA1, HL043PA1, HL053PA1, HL043PA2, HL086PA1, HL056PA1 and HL038PA1) (Figure 2). Interestingly, these were exclusively type I-1a strains belonging to the two clonal clusters CC 3 and CC 31. We PCR-screened our strain collection for the presence of this bacteriocin island, and found it in nine isolates (Table S1, Figure 2), all of which are CC 3 strains. The PCR screen further revealed that all tested strains of the STs 1–4 were PCR-positive for the bacteriocin gene cluster. Interestingly, five out of nine strains were isolated from acne lesions. We concluded that this island exists in a defined subpopulation of *P. acnes*; its biological role and its possible acne-associated significance have to be determined in the future.

### Protospacers within a genomic island of type I *P. acnes* strains encoding a tight adherence (TAD) system

Several spacer sequences matched to another genomic region of type I *P. acnes* strains, which has not been identified previously: 17 spacers (eight unique sequences: no. 6, 7, 8, 10, 12, 17, 22, 23 in Table 1) were detected in CRISPR loci of eight different type II strains. BLAST analyses revealed that the protospacers were located on a large genomic region, which is found exclusively in type I *P. acnes* strains, namely in the partially sequenced genomes of the following 16 HMP strains: HL005PA1, HL007PA1, HL027PA1, HL038PA1, HL043PA1, HL043PA2, HL045PA1, HL046PA2, HL053PA1, HL067PA1, HL072PA1, HL072PA2, HL074PA1, HL078PA1, HL087PA3 and J165 (Figure 2). Within the type I lineage, these strains belong to different CCs (10 strains to CC 3, five strains to CC 18, three strains to CC 28, two strains to CC 31). Again, we PCR-screened our strain collection for the existence of genes located within this island: we found six strains carrying this locus, comprising type I isolates of the CCs 3 and 18 (Table S1, Figure 2). Curiously, also one type II strain (CCUG36609, ST 53), which carries the CRISPR/*cas* locus, was PCR positive for this genetic element.

**Figure 3:**
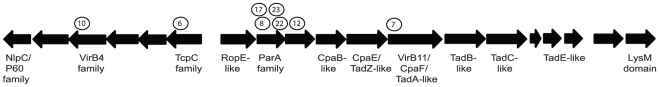
Tight adherence (TAD) gene cluster in *P. acnes* strain 15.1.R1. The gene cluster [GenBank: JQ612072] encoding proteins similar to TadZABC is shown. This cluster of 20.2 kb is part of the 54 kb mobile genetic element in strain *P. acnes* 15.1.R1 and other type I *P. acnes* strains. The annotation is based on sequence similarity, employing the tools BLASTP and InterPro. Genes without annotation are considered hypothetical. CRISPR spacers that are identical or similar to sequences of this mobile genetic element are marked; the numbers refer to Table 1.

**Figure 4:**
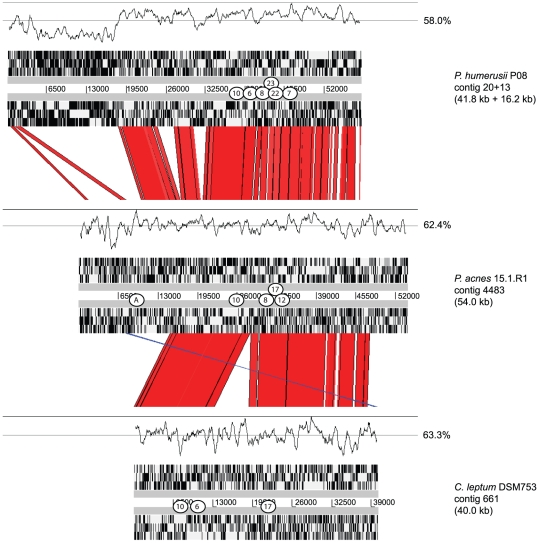
Sequence similarity of TAD locus in strains of *P. acnes*, “*P. humerusii*” and *C. leptum*. A sequence alignment of the partial TAD-like loci of *P. acnes* 15.1.R1, “*P. humerusii*” P08 [GenBank: AFAM00000000] and *C. leptum* DSM753 [GenBank:ABCB00000000] is shown. High sequence similarity (shown in red, >95% nucleotide identity) exists between these loci in different species. The 5′ end of the “*P. humerusii*” contig, which has a lower G+C content, matches to a genomic region in the KPA chromosome (PPA1278–PPA1304). The location of CRISPR spacers is indicated; the protospacer “A” refers to a spacer found in the CRISPR region of *P. avidum*. The G+C content is depicted above the open reading frames (sliding window 500 bp); the percentages refer to the average G+C content of the contigs. The figure was made using the Artemis Comparison Tool (ACT, Sanger).

In order to in-depth analyze this mobile genetic element, strain 15.1.R1 (ST 3), an acne isolate, was selected for sequencing (Table S1). After pyrosequencing, spacer sequences were searched against all contigs of the draft genome. Spacers no. 8, 10, 12 and 17 could be located on one sequence contig of 54 kb [contig4483, GenBank: JQ612072]. No core genome insertion boundaries could be detected at the 5′ and 3′ ends of this contig; it apparently exists as an extrachromosomal element in strain 15.1.R1. Surprisingly, functional analysis of the genes located on this contig revealed that it encodes a secretion system-like complex known as tight adherence (TAD) system (Figure 3, Figure 4). The TAD system has been studied in the Gram-negative *Aggregatibacter actinomycetemcomitans* [22], [23]; the system is dedicated to the assembly and export of Flp pili, and it was shown to be important for host colonization and pathogenesis. Gram-positive Actinobacteria such as *Mycobacterium, Corynebacterium* and *Streptomyces* also possess a TAD system composed of TadZABC [23]; homologs of these four proteins were found on the 54 kb element in strain 15.1.R1. The function of this system in Actinobacteria is so far unexplored. Apart from *tad*-like genes, several other functions are encoded, including toxin/antitoxin systems, a putative autolysin, and ParA family protein, which possibly functions as a plasmid partitioning protein. Interestingly, several protospacer sequences within this element were located in this *parA* gene (Figure S2, Figure 3), which underlines the importance of this gene within the mobile element.

Further analyses revealed that the TAD-like island of type I *P. acnes* is also present in two other species (Figure 4): in the closely related species “*Propionibacterium humerusii*” and in *Clostridium leptum*. The species tentatively designated as *“P. humerusii”* is yet to be effectively and validly described. “*P. humerusii*” strain P08 was isolated from the humeral membrane of a patient who underwent revision of a failed total shoulder arthroplasty [24]. Three other “*P. humerusii*” strains from the HMP (HL037PA2, HL037PA3 and HL044PA1) also contain the TAD locus. The other species positive for this TAD locus is *C. leptum* (strain DSM753), belonging to the phylum *Firmicutes* and a member of a predominant group of bacteria in the gastrointestinal tract [25]. This raises interesting questions about the acquisition and dissemination of this genetic element. In “*P. humerusii*” the TAD locus is encoded on a larger region (Figure 4), which could be inserted into the chromosome; the 5′-end of the island-containing contig20 matches to the region PPA1278–PPA1304 of the KPA genome. However, this region is itself an island-like region in the KPA genome [12], and encodes non-ribosomal peptide synthetases. The region also encodes a ParA family protein with similarities to known plasmid partitioning proteins, indicating that this genomic region might be acquired via plasmid insertion. Thus, it is possible that the TAD-containing region is an extrachromosomal element in *P. acnes* 15.1.R1 as well as in “*P. humerusii*”. The origin of this TAD locus remains unclear. Its G+C content is 63% in all three species (*P. acnes, “P. humerusii”, C. leptum*), which differs strongly from the *C. leptum* backbone genome (50.2%) and slightly from *P. acnes* and *“P. humerusii”* genomes (60%). Thus, it can be ruled out that the TAD locus originated from *C. leptum*. A phylogram tree was constructed based on all available *tadA* sequences (Figure S3). This revealed the higher similarity between *tadA* of *C. leptum* and *P. acnes*, compared to *“P. humerusii”* and *P. acnes*. Thus, the HGT of the TAD locus between *P. acnes* and *C. leptum* must have been a relatively recent event.

Taken together, the data shows that the TAD locus is a mobile genetic element that possibly exists as an extrachromosomal element. In contrast to the bacteriocin island, the TAD locus is disseminated in a rather diverse set of type I *P. acnes* strains; its dissemination even exceeds the species level. Type II strains with TAD locus-specific spacers in their CRISPR region are apparently protected from the acquisition of this locus.

### Protospacers present in other foreign DNA elements

Other spacers were found in CRISPR regions of type II *P. acnes*; three spacers matched to sequences not related to *P. acnes* or phages. Spacer no. 19 is identical to a sequence from a microsatellite sequence of the red algae *Porphyra haitanensis* clone PH-41 and spacer no. 21 is identical to a sequence located on the 271 kb plasmid pACMV1 found in *Acidiphilium multivorum* AIU301, an acidophilic, aerobic, anoxygenic and phototrophic bacterium isolated from pyritic acid mine drainage [26]. Apparently, *P. acnes* was exposed to mobile genetic elements harboring these sequences. If they actually originated from these species is highly unlikely, since they do not share the same ecological niche with *P. acnes*.

Spacer no. 13 matched to a sequence within the *cse3* gene of *Verrucosispora maris* AB-18-032, a marine actinomycete [27]; *cse3* encodes another Cas family protein. That could indicate that CRISPR/*cas*-positive *P. acnes* strains harboring this spacer are protected against the acquisition of an additional CRISPR/*cas* locus. This underlines that *cas* genes containing genetic loci are themselves mobile genetic elements, as many CRISPR/*cas* loci, including the ones found in *P. acnes*, harbor signatures of their horizontal acquisition [28]–[30]; see below).

### Evolutionary aspects: acquisition and deletion of the CRISPR/*cas* locus in *P. acnes*

**Figure 5:**
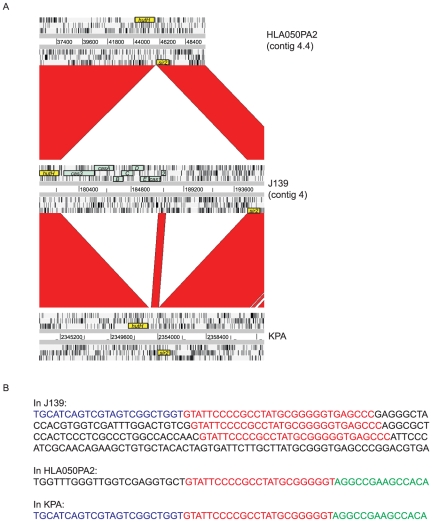
The CRISPR/*cas*-harboring locus is a genomic island in *P. acnes*. (A) A 16 kb gene cluster containing the CRISPR/*cas* locus is inserted as an island-like region in type II *P. acnes* genomes (strain J139) between two genes of the core genome, encoding HutH (histidine ammonia-lyase) and a Sir2 family protein (highlighted in yellow). Some type II strains (shown here: HL050PA2 [GenBank:ADYC00000000]) have no insertion at this genomic location. In contrast, all tested type I strains have *cas* gene fragments (*cas2* and the 3′-end of *cas1*) inserted at this site (shown here: KPA), indicating that a functional CRISPR/Cas system was lost in the evolutionary history of type I strains. Red, >97% nucleotide sequence identity. (B) Deletion of the CRISPR locus and downstream genes in KPA occurred within the first CRISPR repeat sequence (red).

In all analyzed CRISPR/*cas*-positive *P. acnes* genomes the CRISPR/*cas* locus is located on a 16 kb genomic region, which is inserted between genes encoding a histidine ammonia-lyase (HutH = PPA2170 in genome KPA) and a Sir2 family protein (PPA2172) (Figure 5A). The 16 kb CRISPR/*cas*-containing genomic region harbors additional genes downstream of the CRISPR locus, including a putative restriction-modification system. This indicates that resistance mechanisms to control incoming DNA are apparently clustered and have likely been horizontally acquired and inserted into the core genome, as it was suggested for CRISPR/*cas* loci in other bacteria [29]–[31]. Interestingly, there is a genetic fragment in almost all CRISPR/*cas*-negative genomes (i.e. type I strains), composed of *cas2* and the 3′-end of *cas1* (PPA2171 in the KPA genome) (Figure 5A). These *cas* gene fragments of type I strains are identical to the corresponding genes in the complete CRISPR/*cas* loci of type II strains, indicating that the CRISPR/*cas* gene cluster was partially deleted in type I strains. Two deletion events could have taken place, deleting the *cas* genes located on a 8.4 kb fragment (*cas3-casA-casB-casC-casD-casE* and the 5′-end of *cas1*) and the downstream region of *cas2* (7 kb fragment containing CRISPR and downstream genes). A hybrid-like pattern of the CRISPR/*cas*-containing 16 kb locus is supported by the analysis of the respective locus in *Propionibacterium avidum* (see below). Looking closer at the second site of deletion, it occurred within the first copy of the CRISPR repeat (Figure 5B), indicating that the repeats are associated with genomic instability. PCR analyses revealed that a few strains (HL050PA2, 34.1.A1, 39.3.R1 and CCUG33206) did not contain *cas* gene fragments; these were all type II strains (Figure 5A). Among the type II strains one strain (CCUG38203, ST 53) was exceptional, since it contained *cas1/cas2* gene fragments as seen in type I strains.

We hypothesize that type II *P. acnes* strains represent more ancient strains of the species *P. acnes*, compared to type I strains, which is supported by the significantly higher degree of sequence diversity among type II than among type I strains as illustrated in Figure 2. A possible evolutionary scenario would be that an ancestor type II strain without CRISPR/*cas* (similar to HL050PA2, 34.1.A1, 39.3.R1, CCUG33206) has acquired this system. Subsequent (partial) loss of the CRISPR/*cas* system gave rise to a progenitor type I *P. acnes* strain. Conceivably, the deletion of the CRISPR/*cas* island facilitated the acquisition of new genetic material, in particular of the two mobile genetic elements described here, thus led to the genomic repertoire of present day type I *P. acnes* strains associated with acne. ST 53 could constitute a transitional *P. acnes* class between type I and type II, since some ST 53 strains possess markers of type I strains (TAD locus, *cas* gene fragments).

### CRISPRs in *Propionibacterium avidum*

CRISPR loci can be found in the genomes of other propionibacteria. *P. avidum* (strain ATCC 25577) possesses a CRISPR/*cas* region that differs notably from the locus in *P. acnes* genomes. The island is slightly bigger (18.7 kb) compared to the *P. acnes* version. Like in *P. acnes*, the *cas* genes are inserted into the backbone *P. avidum* genome downstream of *hutH*; they are homologous (78% identity on nucleotide level) to the corresponding genes of *P. acnes*. However, the region downstream of CRISPR shows no similarity to the *P. acnes* locus, underlining the hybrid-like nature of the CRISPR/*cas* locus in propionibacteria. The *P. avidium*-specific region encodes an ABC transport system as well as hypothetical proteins. The ABC transport system is similar to macrolide-specific ABC-type efflux carriers (MacAB) [32]. The CRISPR repeat sequence in *P. avidum* is GTCTTCCCCGCCTACGCGGGGGTGAGCC, thus has two nucleotide substitutions compared to the repeat in *P. acnes*. The repeat appears in 28 copies in the *P. avidum* genome. 26 spacers are predicted; they are specific to *P. avidum*. Only four show similarities to known sequences: one is similar to a sequence of the *Pongo abelii* chromosome UNK clone CH276-86H12 (AC188115.1), one to the plasmid pAB510d of *Azospirillum* sp. B510 (AP010950.1), and one to the gene encoding a major facilitator superfamily protein (VAB18032_27756) in *Verrucosispora maris* AB-18-032. Interestingly, one spacer (GCTGAATGGAGAGCGAGCATGAGCACCACCCCT) of the *P. avidum* CRISPR region matches to a sequence within a gene located on the TAD locus-containing element of strain 15.1.R1 (Figure 4). Thus, *P. avidum* was also exposed to the TAD locus-containing mobile element or a related element.

### Conclusions

The CRISPR/Cas system was detected in a subpopulation of *P. acnes* strains; these strains were phylogenetically closely related, all part of the type II lineage of *P. acnes*. Type II strains are often found on healthy skin or in deep tissue infections, they are rarely associated with acne vulgaris [8]. In contrast, no CRISPR/*cas* locus was identified in type I strains. The CRISPR/Cas system apparently protects against siphovirus infections and the acquisition of two mobile genetic elements. These elements encode a bacteriocin synthesis pathway and a putative TAD system, respectively, which might be important fitness functions. These elements were found in a subset of type I *P. acnes* strains, namely in strains belonging to the clonal complexes CC 3, CC 18, CC 28, and CC 31. These CCs, except from CC 28, were previously identified as being associated with acne vulgaris. Moreover, CC 36, a clonal complex within type I, which is not associated with acne but with healthy skin, does not possess these mobile genetic elements. Thus, it will be interesting to study the biological role of these mobile genetic elements with respect to their putative contribution to acne formation and/or progression. However, not all acne-associated *P. acnes* strains harbor these mobile genetic elements. Whereas the bacteriocin-encoding island is restricted to strains of CC 3, the TAD locus-containing element is scattered among diverse type I strains. That could indicate that the locus confers properties to *P. acnes* which are not directly linked to their association with acne. From the evolutionary perspective, the data suggest that CRISPR/*cas*-negative *P. acnes* strains, i.e. type I strains, have lost the CRISPR locus by deletion events, as judged from the presence of remnant *cas* gene fragments in type I strains. This supports the assumption that type I strains are an evolutionary more recent subpopulation of *P. acnes*.

## Materials and Methods

### *P. acnes* strains and their phylogenetic analysis

108 strains of *P. acnes* were included in this study, comprising isolates from acne patients and from controls without skin disease, as well as isolates from opportunistic infections [8]. Most of the latter were retrieved from recognized public collections representing clinical isolates from the United Kingdom, U.S.A., Sweden, Norway, and Germany collected between 1920 and 2004. All strains were sequence typed based on allelic profiles of nine housekeeping gene sequences according to the Aarhus MLST scheme (http://pacnes.mlst.net/) [8]; the collection contained isolates from all known sequence types of *P. acnes*. A phylogenetic tree was constructed based on 4,287-bp concatamer of partial sequences of the nine housekeeping genes using the Minimum evolution algorithm in MEGA version 5.05 [33].

### PCR conditions and sequencing

For preparation of bacterial DNA, a loopfull of bacteria was suspended in 100 µl of double sterilized water. Twenty µl of this solution was mixed with 80 µl 0.05 M NaOH and incubated at 60°C for 45 minutes. Subsequently, 9.2 µl of 1 M Tris-HCl, pH 7.0 were added and the solution was diluted 1∶100. Five µl of this solution was used for PCR. The CRISPR region was PCR-amplified using the following primers: CRISPR-for, 5′-TGATCCTGACGAGGGATACG, and CRISPR-rev, 5′-CTTTGCTTGCGGTACTGGAC. In addition, the presence of *cas*1 was verified by PCR using the primers cas1-for 5′-TCGAGCACTGCATTGTCAAC and cas1-rev 5′-CGTATCCCTCGTCAGGATCA. The presence or absence of *cas1/cas2* gene fragments in the core genome between the genes *hutH* and *sir2* was tested by PCR in all type I and II *P. acnes* strains using the primers hutH, 5′-CGCGGAGTACATGCTCAGTC and sir2, 5′-GTCGACTCCGGGCATATGAT. The PCR product was 1.94 kb for strains with *cas1/cas2* fragments and 1.22 kb for strains without fragments.

A region (genes HMPREF0675_3182 and HMPREF0675_3182 in the genome of *P. acnes* SK137) within the bacteriocin locus was PCR-amplified with the primers: bcn-for, 5′-CACTCAGGTACCCCGGTGA and bcn-rev, 5′-CAAGGCGTTTCTCGAGTGC. To check for the presence of the TAD locus, three genes (*tadZ/cpaE*-like, *tadA/cpaF/virB11*-like and *tadB*-like genes) were PCR amplified with the primers tadZ-for, 5′-GTCGAGGACGCCGACATGA, tadZ-rev, 5′-GATTTCGAACAGCGGCTG, tadA-for, 5′-GGGTAACCATGAGGTGGTTG, tadA-rev, 5′-CTGAACGGATCTGCTCAACA, tadB-for, 5′-TTGGAGACCGATCAGGAG and tadB-rev, GAGATCCGCACCGCTGTGA. It was ascertained that the primer sequences were conserved in the bacteriocin or TAD locus-positive *P. acnes* genomes from the HMP. The hotmastermix (Eppendorf) was used as polymerase. The temperature profile for all PCRs was an initial denaturation at 96°C for 40 s, followed by 35 cycles at 94°C for 35 s, 55°C for 40 s, and 72°C for 1 min, followed by a final extension at 72°C for 7 min. Amplicons were purified using Wizard Minicolumns (Promega). Sequencing of both strands of the amplified CRISPR-containing fragments was achieved with the CRISPR-for/rev primers. Sequences were deposited in GenBank, with accession numbers JQ287501-JQ287524 (see Table S1).

### Sequence analyses and software tools

To identify CRISPR regions we used the CRISPR finder tool (http://crispr.u-psud.fr). Spacer sequences were compared to the internal spacer database by blastn. In addition, all spacers were searched against the NR database and the microbial genome database of NCBI. Up to three mismatches were allowed between subject and query sequence. Genome and sequence contig comparisons were done using ACT (http://www.sanger.ac.uk/resources/software/act/) [34] and WebACT (http://www.webact.org/).

Draft sequencing of the genome of *P. acnes* strain 15.1.R1 was performed at the Institute for Genome Sciences, University of Maryland School of Medicine, Baltimore, USA, using the 454 FLX Titanium pyrosequencing technology. The draft genome was assembled into contigs using Celera Assembler v6.1. Contigs were blast-searched for loci containing protospacer sequences identified in our screen. Protospacers were only found in contig4483, designated TAD locus. This contig was annotated using the RAST tool [35]. The sequence of contig4483 was deposited in GenBank (accession number JQ612072).

Besides the five closed *P. acnes* genomes (strain SK137, CP001977.1; strain 266, CP002409.1; strain KPA171202, AE017283.1; strain 6609, CP002815.1; strain ATCC11828, CP003084.1), 64 *P. acnes* draft genomes deposited in GenBank were searched for CRISPR, bacteriocin and TAD loci. These are reference genomes for the HMP (http://www.ncbi.nlm.nih.gov/bioproject/51439). They were sequenced at the Genome Sequencing Center at Washington University, School of Medicine (60 strains) and at the J. Craig Venter Institute (four strains). Accession numbers are, ADJL00000000, strain J165; AFUM00000000, strain SK 182; ADJM00000000, strain SK187; ADFS00000000, strain J139; PRJNA49245, strain HL001PA1; PRJNA49265, strain HL002PA1; PRJNA49267, strain HL002PA2; PRJNA49269, strain HL002PA3; PRJNA49225, strain HL005PA1; PRJNA49227, strain HL005PA2; PRJNA49229, strain HL005PA3; PRJNA49231, strain HL005PA4; PRJNA49271, strain HL007PA1; PRJNA49169, strain HL013PA1; PRJNA49171, strain HL013PA2; PRJNA49161, strain HL020PA1; PRJNA49211, strain HL025PA1; PRJNA49213, strain HL025PA2; PRJNA49257, strain HL027PA1; PRJNA49259, strain HL027PA2; PRJNA49241, strain HL030PA1; PRJNA49243, strain HL030PA2; PRJNA49247, strain HL036PA1; PRJNA49249, strain HL036PA2; PRJNA49251, strain HL036PA3; PRJNA49279, strain HL037PA1; PRJNA49281, strain HL037PA2; PRJNA49283, strain HL037PA3; PRJNA49203, strain HL038PA1; PRJNA49175, strain HL043PA1; PRJNA49177, strain HL043PA2; PRJNA49253, strain HL044PA1; PRJNA49167, strain HL045PA1; PRJNA49221, strain HL046PA1; PRJNA49223, strain HL046PA2; PRJNA49233, strain HL050PA1; PRJNA49237, strain HL050PA2; PRJNA49239, strain HL050PA3; PRJNA49163, strain HL053PA1; PRJNA49165, strain HL053PA2; PRJNA49273, strain HL056PA1; PRJNA49215, strain HL059PA1; PRJNA49217, strain HL059PA2; PRJNA49201, strain HL060PA1; PRJNA49261, strain HL063PA1; PRJNA49263, strain HL063PA2; PRJNA49255, strain HL067PA1; PRJNA49179, strain HL072PA1; PRJNA49181, strain HL072PA2; PRJNA49183, strain HL074PA1; PRJNA49173, strain HL078PA1; PRJNA49275, strain HL082PA1; PRJNA49277, strain HL082PA2; PRJNA49207, strain HL083PA1; PRJNA49209, strain HL083PA2; PRJNA49219, strain HL086PA1; PRJNA49195, strain HL087PA1; PRJNA49197, strain HL087PA2; PRJNA49199, strain HL087PA3; PRJNA49205, strain HL092PA1; PRJNA49187, strain HL110PA1; PRJNA49189, strain HL110PA2; PRJNA49191, strain HL110PA3; PRJNA49193, strain HL110PA4

### Ethics

The study protocol was approved by the Ethics Committee of the County of Aarhus, and the study was conducted according to the principles of the declaration of Helsinki. Written informed consent was obtained from study participants and/or their legal guardians.

## Supporting Information

Figure S1 **Bacteriocin island in** ***P. acnes*** **strain SK137 [GenBank: CP001977.1].** The spacer sequence can be found in the 3′end of the island (black arrow). The island contains a gene cluster for bacteriocin synthesis. Several genes are homologous to the Sag gene cluster for streptolysin S synthesis in *Streptococcus pyogenes*. SagD is a scaffold or docking protein, SagC is a cyclodehydratase and SagB participates in the maturation of streptolysin S from a ribosomally produced precursor polypeptide. The Abi genes are probably involved in self-immunity. The cluster is inserted into the backbone genome, within a gene encoding a ATP-dependent helicase (PPA0155 in KPA, upper genome).
(PDF)

Figure S2 **Location of protospacers in the** ***parA*** **gene of the TAD locus.** One gene within the TAD locus harbors 4 protospacer sequences. This gene encodes a putative plasmid partitioning protein (Soj/ParA family protein). 3 regions (in yellow) in the coding strand of *parA* in “*P. humerusii*” P08 (P.hum) are identical to spacers no. 8, 22, 23. An alignment of *parA* of “*P. humerusii*” and of *P. acnes* 15.1.R1 is shown. There is one additional protospacer (no. 17, in cyan) in the antisense strand of the *parA* gene of *P. acnes* 15.1.R1 (with one mismatch).
(PDF)

Figure S3 **Phylogenetic tree of** ***tadA*** **of** ***P. acnes*** **, “** ***P. humerusii*** **” and** ***C. leptum*** **.** Only complete *tadA* sequences were taken into consideration. The tree reveals a high similarity of *tadA* of *C. leptum* and *P. acnes* (genetic distance 0.012), indicative of a recent HGT event. In contrast, the genetic distance between *tadA* of *“P. humerusii”* and *P. acnes* is 0.149 (+/−0.003) and reflects the overall genetic diversification (0.141+/−0.0006 based on the nine genes used in the MLST scheme) of the two species since they separated, i.e. the *tadA* gene has been present in a common ancestor.
(EPS)

Table S1 Strains used in this study and PCR results for TAD, bacteriocin (BCN) and CRISPR loci. Listed are also the GenBank accession numbers of CRISPR containing sequences.
(DOC)

Table S2 **Complete list of spacer sequences identified in type II strains of** ***P. acnes*** **.**
(DOC)
