# Supplementary material for: CRISPR/cas Loci of Type II Propionibacterium acnes Confer Immunity against Acquisition of Mobile Elements Present in Type I P. acnes
Source: PLoS One. 2012 Mar 30;7(3):e34171. doi: 10.1371/journal.pone.0034171 (PMC3316620; doi:10.1371/journal.pone.0034171)
Supplement: Figure S1 — Bacteriocin island in P. acnes strain SK137 [GenBank: CP001977.1]. The spacer sequence can be found in the 3′end of the island (black arrow). The island contains a gene cluster for bacteriocin synthesis. Several genes are homologous to the Sag gene cluster for streptolysin S synthesis in Streptococcus pyogenes. SagD is a scaffold or docking protein, SagC is a cyclodehydratase and SagB participates in the maturation of streptolysin S from a ribosomally produced precursor polypeptide. The Abi genes are probably involved in self-immunity. The cluster is inserted into the backbone genome, within a gene encoding a ATP-dependent helicase (PPA0155 in KPA, upper genome). (PDF) [file pone.0034171.s001.pdf]

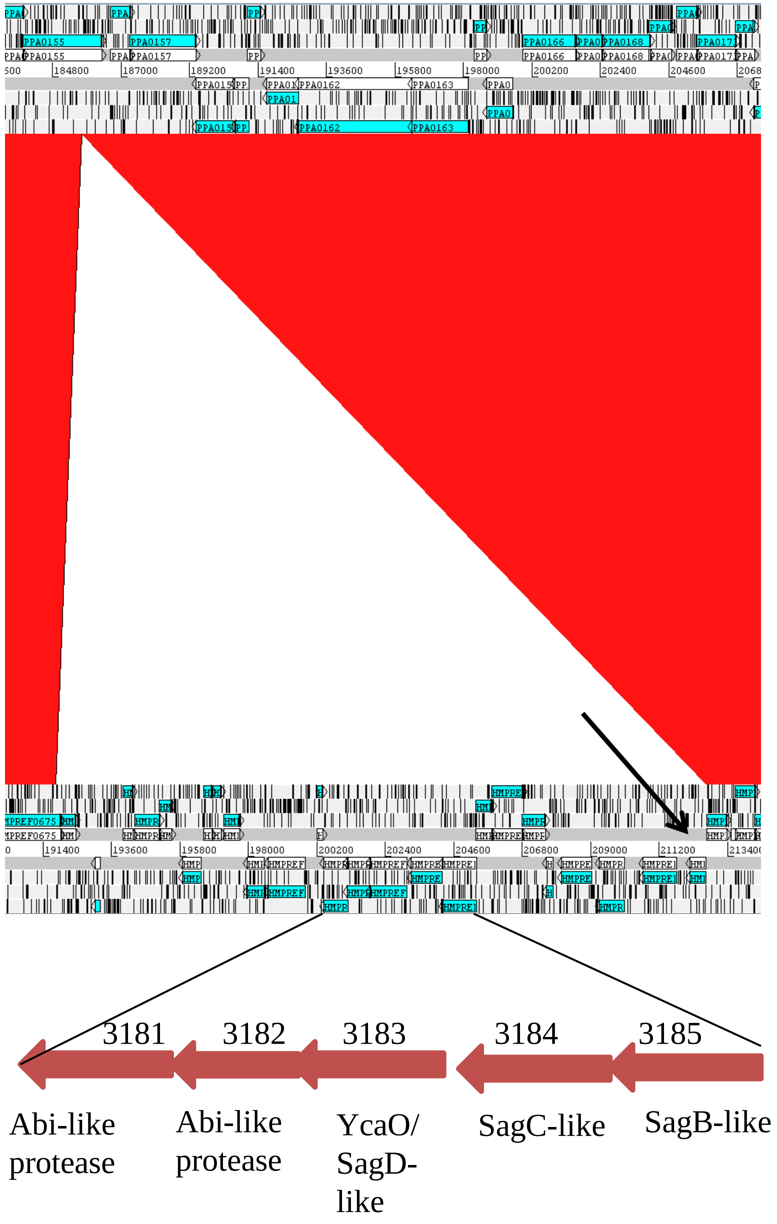

Figure S1. Bacteriocin island in *P. acnes* strain SK137

The spacer sequence can be found in the 3'end of the island (black arrow). The island contains a gene cluster for bacteriocin synthesis. Several genes are homologous to the Sag gene cluster for streptolysin S synthesis in *Streptococcus pyogenes*. SagD is a scaffold or docking protein, SagC is a cyclodehydratase and SagB participates in the maturation of streptolysin S from a ribosomally produced precursor polypeptide. The Abi genes are probably involved in self-immunity. The cluster is inserted into the backbone genome, within a gene encoding a ATP-dependent helicase (PPA0155 in KPA, upper genome).
