# Supplementary material for: CRISPR/cas Loci of Type II Propionibacterium acnes Confer Immunity against Acquisition of Mobile Elements Present in Type I P. acnes
Source: PLoS One. 2012 Mar 30;7(3):e34171. doi: 10.1371/journal.pone.0034171 (PMC3316620; doi:10.1371/journal.pone.0034171)
Supplement: Figure S2 — Location of protospacers in the parA gene of the TAD locus. One gene within the TAD locus harbors 4 protospacer sequences. This gene encodes a putative plasmid partitioning protein (Soj/ParA family protein). 3 regions (in yellow) in the coding strand of parA in “P. humerusii” P08 (P.hum) are identical to spacers no. 8, 22, 23. An alignment of parA of “P. humerusii” and of P. acnes 15.1.R1 is shown. There is one additional protospacer (no. 17, in cyan) in the antisense strand of the parA gene of P. acnes 15.1.R1 (with one mismatch). (PDF) [file pone.0034171.s002.pdf]

**Figure S2. Location of protospacers in the *parA* gene of the TAD locus**

One gene within the TAD locus harbors 4 protospacer sequences. This gene encodes a putative plasmid partitioning protein (Soj/ParA family protein). 3 regions (in yellow) in the coding strand of *parA* in "*P. humerusii*" P08 (P.hum) are identical to spacers no. 8, 22, 23. An alignment of *parA* of "*P. humerusii*" and of *P. acnes* 15.1.R1 is shown. There is one additional protospacer (no. 17, in cyan) in the antisense strand of the *parA* gene of *P. acnes* 15.1.R1 (with one mismatch).

```
P.hum  1     ATGACCTCGACCAGACCGATTGACCGATCCCCGTTCGACCGCACCATCGCGGTATGGAAC  60
             |||||||||||||||||||||||||||||||||||||||||||| || ||||| ||||||
151R1  1     ATGACCTCGACCAGACCGATTGACCGATCCCCGTTCGACCGCACTATTGCGGTGTGGAAC  60

P.hum  61    CACAAAGGCGGCACGTTCAAGACGTCCGTCGTGGCGAACCTGGGATACCTGTTCGCCGCC  120
             ||||||||||||||||||||||||||||||||||||||||||||||||||||||||||||
151R1  61    CACAAAGGCGGCACGTTCAAGACGTCCGTCGTGGCGAACCTGGGATACCTGTTCGCCGCC  120

P.hum  121   GGTGGCAACAAGGTGCTGATGGTCGACATGGACCCCCAAGCCAATCTCGACATTGACTTC  180
             |||||||||||||||||| ||||||||||||||||||| |||||||||||||||||||||
151R1  121   GGTGGCAACAAGGTGCTGCTGGTCGACATGGACCCCCAGGCCAATCTCGACATTGACTTC  180   Spacer no. 17

P.hum  181   GGTATACCCGCGGGAGAACGTGAACGGGGCATGGGATTAGCCGAGGCGCTACGTGAGGGG  240   Spacer no. 8
             ||||||||||||||||||||||||||||||||||||||||||||||||||||||||||||
151R1  181   GGTATACCCGCGGGAGAACGTGAACGGGGCATGGGATTAGCCGAGGCGCTACGTGAGGGG  240

P.hum  241   ACGGCCCTTCCCCCACCGC-GTCATCTCAGCGAAAACCTTCACCTGGTCAGTGGCGGCCC  299
             ||||||||||||||||||| | |||||||||||||||||||||||||||||||||||| |
151R1  241   ACGGCCCTTCCCCCACCGCAG-CATCTCAGCGAAAACCTTCACCTGGTCAGTGGCGGCGC  299

P.hum  300   GGCCCTCCACGAGTTCACCGACCCCGCCTCCCTAGCGGCCATCCTCGAGCGGGTCACTAC  359
              || ||| ||||||||||||||||||| ||| |||||||||||||||| || ||||| ||
151R1  300   TGCTCTCAACGAGTTCACCGACCCCGCATCCTTAGCGGCCATCCTCGACCGAGTCACCAC  359

P.hum  360   CGCACGCTACGACCTGCTGGCCCAGGCCCTCGCACCGCTGGCCTGGGACTACGACCTTAT  419
             |||||||||||||||||| || |||||||| || ||||||||||||||||| ||||| ||
151R1  360   CGCACGCTACGACCTGCTAGCTCAGGCCCTTGCCCCGCTGGCCTGGGACTATGACCTCAT  419

P.hum  420   CCTT-ATCGATTCCGGGCCGGCCCAGACTGTGCTGTCCCAGACCATCCTTGGAGTAGCCC  478
             | || ||||| ||||||||||| || ||| ||||||||||||||||||||||||||||||
151R1  420   C-TTCATCGACTCCGGGCCGGCACAAACTCTGCTGTCCCAGACCATCCTTGGAGTAGCCC  478

P.hum  479   GTTACCTGGTGGTGCCCACCCGCTCCGATAACGCCTCGATCACCGGGCTGGTCGGCGTTC  538   Spacer no. 23
             | |  ||||| |||||||||||| ||||||||||||| ||||| || || |||| |||||
151R1  479   GCTGGCTGGTAGTGCCCACCCGCACCGATAACGCCTCAATCACTGGCCTCGTCGACGTTC  538

P.hum  539   AGGACGCCATCGATGAGGTCGCCGACTGCAACCCCGGCCTG-AACCTGCTCGGTGTTGTG  597
             | ||||||||||| | ||| |||  ||||||||| | |||  ||| | ||||| |||||
151R1  539   AAGACGCCATCGACGGGGTTGCCTCCTGCAACCCTGACCTTCAAC-TACTCGGCGTTGTC  597

P.hum  598   CTGGCTGGTGTGGGCACTCAGGCCACCCGGATCGGGGCCGATAAACGCAAGGCCATTGAC  657
             || || || |||||  | | ||||||||||||||  || ||||||||| | ||||| |||
151R1  598   CTAGCCGGCGTGGGGGCCCGGGCCACCCGGATCGCCGCAGATAAACGCCACGCCATCGAC  657

P.hum  658   ACGGT-CTTGGGGG-AGGGGACCGTCTTCGAGGCGATCATTCACTACTCCGAAAAAGTGG  715
             || || || ||||| |||  ||||| ||||| ||| |||| ||||||||||| || |||
151R1  658   ACAGTGCT-GGGGGCAGGA-ACCGTTTTCGATGCGGTCATCCACTACTCCGAGAAGGTGT  715

P.hum  716   CCGTGCTCGCACGCCAGCAGGGTAAGACCGTCGCCGAGTTGGCTGGCGAATACCACCACA  775
             |||||||||||||||||||||| ||||||||||||||| | ||   ||| |||||| |||
151R1  716   CCGTGCTCGCACGCCAGCAGGGCAAGACCGTCGCCGAGCTAGCCAACGAGTACCACAACA  775

P.hum  776   CCCAGCCCGCCTACACCTACCTAGCCCAGGGCAAAAAGGTTCCCAACGTCGCTAAGGCGG  835
             |||||||||||||||||||||||||  | ||| | ||  | ||||||||||| ||||| |
151R1  776   CCCAGCCCGCCTACACCTACCTAGCTAAAGGCCAGAACATCCCCAACGTCGCCAAGGCAG  835

P.hum  836   CCGTC-GCCATTGAAACCGACTACCT-ACGGTTGGCCACCGAAATCAGTGACCGCATGTT  893   Spacer no. 22
             ||||| || || ||||| || || || | || |||||||||| ||||| |||||||||||
151R1  836   CCGTCAGC-ATCGAAACTGATTATCTGA-GGCTGGCCACCGAGATCAGCGACCGCATGTT  893

P.hum  894   CGCCGGCGACGAGCAGGAG  912
             | || ||||||||||||||
151R1  894   CACCAGCGACGAGCAGGAG  912
```
