# Supplementary material for: CRISPR/cas Loci of Type II Propionibacterium acnes Confer Immunity against Acquisition of Mobile Elements Present in Type I P. acnes
Source: PLoS One. 2012 Mar 30;7(3):e34171. doi: 10.1371/journal.pone.0034171 (PMC3316620; doi:10.1371/journal.pone.0034171)
Supplement: Table S1 — Strains used in this study and PCR results for TAD, bacteriocin (BCN) and CRISPR loci. Listed are also the GenBank accession numbers of CRISPR containing sequences. (DOC) [file pone.0034171.s004.doc]

**Table S1. Strains used in this study and PCR results for Tight adherence (TAD), Bacteriocin (Bcn) and CRISPR loci**

| strain | ST | Disease | TAD | BCN | CRISPR locus GenBank accession number |
| --- | --- | --- | --- | --- | --- |
| 42.1.R1 | 1 | Healthy skin |  | + |  |
| 37.1.R1 | 2 | Acne severe | + | + |  |
| 3.1.A1 | 3 | Healthy skin | + | + |  |
| CCUG 48370 | 3 | Purulent vaginal discharge |  | + |  |
| 15.2.A1 | 3 | Healthy skin | + | + |  |
| 25.1.L1 | 3 | Acne mild |  | + |  |
| 29.1.L1 | 3 | Acne mild |  | + |  |
| 15.1.R1 | 3 | Acne | + | + |  |
| 19.1.L1 | 4 | Acne moderate |  | + |  |
| 14.1.L1 | 5 | Acne mild |  |  |  |
| CCUG 50480 | 6 | Endocarditis |  |  |  |
| 40.1.R1 | 7 | Acne mild |  |  |  |
| China 7.1 | 8 | Healthy skin |  |  |  |
| China 4.1 | 9 | Healthy skin |  |  |  |
| China 8.1 | 10 | Healthy skin |  |  |  |
| 34.2.R1 | 11 | Acne mild |  |  |  |
| 33.1.A1 | 12 | Acne mild |  |  |  |
| 37.1.L1 | 13 | Acne severe |  |  |  |
| 40.1.L1 | 14 | Acne mild |  |  |  |
| 20.2.A1 | 15 | Acne mild |  |  |  |
| 21.1.A1 | 16 | Healthy skin | + |  |  |
| 26.2.A1 | 17 | Acne moderate |  |  |  |
| 1.4.L1 | 18 | Healthy skin |  |  |  |
| NTCC 737 | 18 | Facial acne |  |  |  |
| CCUG 1794 | 18 | Facial acne |  |  |  |
| CCUG 34938 | 19 | Bacteremia |  |  |  |
| 38.1.R1 | 18 | Acne mild |  |  |  |
| 5.1.A1 | 18 | Healthy skin |  |  |  |
| 20.1.A1 | 18 | Acne severe |  |  |  |
| 3.6.L1 | 18 | Healthy skin |  |  |  |
| 23.1.A1 | 18 | Healthy skin |  |  |  |
| 13.1.A1 | 18 | Acne severe |  |  |  |
| 37.1.A1 | 18 | Acne severe |  |  |  |
| 2.4.L1 | 18 | Healthy skin |  |  |  |
| 13.1.R1 | 18 | Acne severe |  |  |  |
| 6.2.A1 | 18 | Healthy skin |  |  |  |
| 41.1.L1 | 18 | Healthy skin |  |  |  |
| 12.1.A1 | 18 | Acne severe |  |  |  |
| 32.1.R1 | 18 | Healthy skin |  |  |  |
| 37.2.L1 | 18 | Acne mild |  |  |  |
| 12.1.R1 | 20 | Acne severe |  |  |  |
| 4.1.A1 | 20 | Acne mild |  |  |  |
| 19.1.R1 | 21 | Acne moderate |  |  |  |
| 4.4.L1 | 22 | Acne mild |  |  |  |
| 18.1.R1 | 23 | Healthy skin |  |  |  |
| 4.4.R1 | 24 | Acne mild |  |  |  |
| 23.1.L1 | 25 | Healthy skin |  |  |  |
| China 2.1 | 26 | Healthy skin |  |  |  |
| 1.5.L1 | 27 | Healthy skin |  |  |  |
| 2.1.A2 | 27 | Healthy skin |  |  |  |
| CCUG 38453 | 27 | Bone infection |  |  |  |
| 3.2.L1 | 27 | Healthy skin |  |  |  |
| 25.1.R1 | 28 | Healthy skin |  |  |  |
| 27.1.R1 | 29 | Acne mild |  |  |  |
| 15.2.L1 | 29 | Healthy skin |  |  |  |
| 20.2.R1 | 30 | Acne mild |  |  |  |
| 3.6.A1 | 31 | Healthy skin |  |  |  |
| 16.2.R1 | 32 | Acne mild |  |  |  |
| CCUG32901 | 33 | Bacteremia |  |  |  |
| CCUG 47251 | 33 | Foreign body infection |  |  |  |
| DSM16379 | 34 | Normal skin |  |  |  |
| 2.3.A1 | 35 | Healthy skin |  |  |  |
| 21.1.L1 | 36 | Healthy skin |  |  |  |
| CCUG 48138 | 36 | Joint infection |  |  |  |
| 21.2.A1 | 37 | Healthy skin |  |  |  |
| 27.1.A1 | 38 | Acne mild |  |  |  |
| 18.2.A1 | 39 | Healthy skin |  |  |  |
| 27.1.L1 | 40 | Acne mild |  |  |  |
| 36.1.L1 | 41 | Acne mild |  |  |  |
| CCUG 36661 | 42 | Bacteremia |  |  |  |
| CCUG 35900 | 43 | Biopsy |  |  | JQ287514 |
| CCUG 35749 | 43 | Foreign body infection |  |  | JQ287522 |
| CCUG 35547 | 44 | Oliocranian bursitis |  |  | JQ287517 |
| CCUG 36986 | 44 | Wound |  |  | JQ287503 |
| 36.1.R1 | 45 | Acne mild |  |  | JQ287512 |
| CCUG 50655 | 46 | Mandibular gland |  |  | JQ287516 |
| 18.2.L1 | 47 | Healthy skin |  |  | JQ287504 |
| CCUG 33951 | 48 | Blood/bacteremia |  |  | JQ287519 |
| China 2.3 | 49 | Healthy skin |  |  | JQ287524 |
| 7.1.L1 | 50 | Acne mild |  |  | JQ287501 |
| CCUG 27534 | 51 | Urinary tract infection |  |  | JQ287523 |
| 5.1.R1 | 52 | Healthy skin |  |  | JQ287502 |
| CCUG 6369 | 52 | Subcutaneous abscess |  |  | JQ287520 |
| CCUG 6528 | 52 | Acne |  |  | JQ287513 |
| 1.4.R1 | 52 | Healthy skin |  |  | JQ287506 |
| 10.1.R1 | 52 | Healthy skin |  |  | JQ287505 |
| 16.1.A1 | 52 | Acne mild |  |  |  |
| CCUG 37286 | 52 | Blood/bacteremia |  |  | JQ287515 |
| CCUG 38293 | 52 | n.d. |  |  | JQ287518 |
| 18.1.A1 | 53 | Healthy skin |  |  | JQ287508 |
| CCUG 33950 | 53 | Meningitis/CSF |  |  | JQ287521 |
| CCUG 36609 | 53 | Human pustules | + |  | JQ287509 |
| 25.1.A1 | 53 | Acne mild |  |  | JQ287510 |
| 17.1.A1 | 53 | Healthy skin |  |  | JQ287507 |
| CCUG 38203 | 53 | Bacteremia |  |  |  |
| 34.1.A1 | 54 | Acne moderate |  |  |  |
| CCUG 45436 | 55 | Oral cavity |  |  | JQ287511 |
| 39.3.R1 | 56 | Acne mild |  |  |  |
| CCUG 33206 | 57 | Bacteremia |  |  |  |
