# Supplementary material for: CRISPR/cas Loci of Type II Propionibacterium acnes Confer Immunity against Acquisition of Mobile Elements Present in Type I P. acnes
Source: PLoS One. 2012 Mar 30;7(3):e34171. doi: 10.1371/journal.pone.0034171 (PMC3316620; doi:10.1371/journal.pone.0034171)
Supplement: Table S2 — Complete list of spacer sequences identified in type II strains of P. acnes . (DOC) [file pone.0034171.s005.doc]

**Table S2: Complete list of spacer sequences identified in type II strains of *P. acnes***

| **Strain** | **ST** | **source** | **Spacer sequence** | **#** | **BLAST result** | **comment** |
| --- | --- | --- | --- | --- | --- | --- |
| 36.1.R1 | 45 | Acne mild | CGGCCTGCGGCAGATTTTTGTTGCGTTGAATCC | 1 | phages PAS50, PAD20, PA6 |  |
|  |  |  | CGGGCAGAGGATGTGTTGCTCGTTCCTGGATGG | 2 | phages PAS50, PAD20, PA6 |  |
|  |  |  | GTTACGCTGGAACCCCCAATGAACACGCGAGAA | 3 | phages PAD20, PAD42, PAD9, PAS40,etc |  |
|  |  |  | GAGGGCTACCACGTGGTCGATTTGGACTGTCG | 4 | *P. acnes* SK137 | bacteriocin locus |
|  |  |  | CAGGCGCTCCACTCCCTCGCCCTGGCCACCAAC | 5 | No hit |  |
| CUG50655 | 46 | Mandibular gland | AGGGCTACCACGTGGTCGATTTGGACTGTCG | 4 | *P. acnes* SK137 | bacteriocin locus |
|  |  |  | GGCGCTCCACTCCCTCGCCCTGGCCACCAAC | 5 | No hit |  |
| 18.2.L1 | 47 | Healthy skin | ACCGGGCCCATCCCGGTCGGCCTCCTGAAAGG | 6 | type I *P. acnes* genomes | TAD locus |
|  |  |  | TGGCTAGTACGGCCACGGATGAGATTGAGGCC | 7 | type I *P. acnes* genomes | TAD locus |
|  |  |  | GTGAACGGGGCATGGGATTAGCCGAGGCGCTA | 8 | type I *P. acnes* genomes | TAD locus |
|  |  |  | ACCACTCGGGGTGGGACTGCCCAGTTTTATTG | 9 | No hit |  |
|  |  |  | GTGAACGGGGCATGGGATTAGCCGAGGCGCTA | 8 | type I *P. acnes* genomes | TAD locus |
|  |  |  | ACCACTCGGGGTGGGACTGCCCAGTTTTATTG | 9 | No hit |  |
|  |  |  | GCCTACCGTCAGCTGACTCACGCCTCCGCGTT | 10 | type I *P. acnes* genomes | TAD locus |
|  |  |  | TCACACCAGTCATCAGCGTCATAGTCCTCTCGG | 11 | No hit |  |
| CCUG33951 | 48 | Blood | CCATGAGCGGCTGCGCTCCCGATCGGCGGCG | 12 | type I P. acnes genomes | TAD locus |
|  |  |  | GGCGCTCCACTCCCTCGCCCTGGCCACCAAC | 5 | No hit |  |
| 7.1.L1 | 50 | Acne mild | GAGGGCTACCACGTGGTCGATTTGGACTGTCG | 4 | *P. acnes* SK137 | bacteriocin locus |
|  |  |  | AGGCGCTCCACTCCCTCGCCCTGGCCACCAAC | 5 | No hit |  |
| CCUG27534 | 51 | Urinary tract | GGCGCTCCACTCCCTCGCCCTGGCCACCAAC | 5 | No hit |  |
| 5.1.R1 | 52 | Healthy skin | GAGGGCTACCACGTGGTCGATTTGGACTGTCG | 4 | *P. acnes* SK137 | bacteriocin locus |
|  |  |  | AGGCGCTCCACTCCCTCGCCCTGGCCACCAAC | 5 | No hit |  |
| 10.1.R1 | 52 | Healthy skin | TTGGGTGGGTGAGGTCGGGTCGTCAGTCATGAG | 13 | *Verrucosispora maris* AB-18-032 | *cse3* |
|  |  |  | ACGTCGTGAACGTACCCCTTGACGGAGACGGCA | 14 | No hit |  |
|  |  |  | CGGTGTTAACGGCTTGCCTGGCTTGGATGGAGC | 15 | No hit |  |
|  |  |  | CCCATACTGTGCGGGTTGGCGACTATCTGTGGA | 16 | No hit |  |
| 1.4.R1 | 52 | Healthy skin | TTGGGTGGGTGAGGTCGGGTCGTCAGTCATGAG | 13 | *Verrucosispora maris* AB-18-032 | *cse3* |
|  |  |  | GTCGATGTCGAGATTGGCCTGGGGGTCCATGTC | 17 | type I *P. acnes* genomes | TAD locus |
|  |  |  | ACGTCGTGAACGTACCCCTTGACGGAGACGGCA | 14 | No hit |  |
| CCUG6528 | 52 | Acne | CCAGACAACCTCGACAACCTGTTCAGGGGATG | 18 | phage PAS50 |  |
|  |  |  | ATGGCTAGCCCGGATTTTTGGCTGCCTGAGCG | 19 | *Porphyra haitanensis* PH-41 | microsatellite sequences |
|  |  |  | GGGCAGAGGATGTGTTGCTCGTTCCTGGATGG | 2 | phages PAS50, PAD20, PA6 |  |
|  |  |  | TTACGCTGGAACCCCCAATGAACACGCGAGAA | 3 | phages PAD20, PAD42, PAD9, PAS40,etc |  |
|  |  |  | GAGGGCTACCACGTGGTCGATTTGGACTGTCG | 4 | *P. acnes* SK137 | bacteriocin locus |
|  |  |  | CAGGCGCTCCACTCCCTCGCCCTGGCCACCAAC | 5 | No hit |  |
| CCUG37286 | 52 | Blood | GTCGACCAGACCCGGATCGGGCGTTTAGGTCG | 20 | No hit |  |
|  |  |  | CCATACTGTGCGGGTTGGCGACTATCTGTGGA | 16 | No hit |  |
| CCUG38293 | 52 | n.d. | AGGGCTACCACGTGGTCGATTTGGACTGTCG | 4 | *P. acnes* SK137 | bacteriocin locus |
|  |  |  | GGCGCTCCACTCCCTCGCCCTGGCCACCAAC | 5 | No hit |  |
| CCUG6369 | 52 | Abscess | ATCTGCCAACGAGCGAGAGTGGCGCGGTGTTC | 21 | *Acidiphilium multivorum* AIU301 | plasmid pACMV1 |
|  |  |  | GAGGGCTACCACGTGGTCGATTTGGACTGTCG | 4 | *P. acnes* SK137 | bacteriocin locus |
|  |  |  | AGGCGCTCCACTCCCTCGCCCTGGCCACCAAC | 5 | No hit |  |
| 17.1.A1 | 53 | Healthy skin | AGGCGCTCCACTCCCTCGCCCTGGCCACCAAC | 5 | No hit |  |
| 18.1.A1 | 53 | Healthy skin | ACCGGGCCCATCCCGGTCGGCCTCCTGAAAGG | 6 | type I *P. acnes* genomes | TAD locus |
|  |  |  | TGGCTAGTACGGCCACGGATGAGATTGAGGCC | 7 | type I *P. acnes* genomes | TAD locus |
|  |  |  | GTGAACGGGGCATGGGATTAGCCGAGGCGCTA | 8 | type I *P. acnes* genomes | TAD locus |
|  |  |  | ACCACTCGGGGTGGGACTGCCCAGTTTTATTG | 9 | No hit |  |
|  |  |  | GTGAACGGGGCATGGGATTAGCCGAGGCGCTA | 8 | type I *P. acnes* genomes | TAD locus |
|  |  |  | ACCACTCGGGGTGGGACTGCCCAGTTTTATTG | 9 | No hit |  |
|  |  |  | GCCTACCGTCAGCTGACTCACGCCTCCGCGTT | 10 | type I *P. acnes* genomes | TAD locus |
|  |  |  | (T)CACACCAGTCATCAGCGTCATAGTCCTCTCGG | 11 | No hit |  |
| CCUG36609 | 53 | Pustules | CACCGGGCCCATCCCGGTCGGCCTCCTGAAA | 6 | type I P. acnes genomes | TAD locus |
|  |  |  | TCACACCAGTCATCAGCGTCATAGTCCTCTC | 11 | No hit |  |
| 25.1.A1 | 53 | Acne mild | CGACTACCTACGGTTGGCCACCGAAATCAGTG | 22 | type I *P. acnes* genomes | TAD locus |
|  |  |  | GCCTCGATCACCGGGCTGGTCGGCGTTCAGGA | 23 | type I *P. acnes* genomes | TAD locus |
|  |  |  | TGCGCTGTAGACATGATCATTCCCCCGCTCTC | 24 | No hit |  |
|  |  |  | TGCGCTGTAGACATGATCATTCCCCCGCTCTC | 24 | No hit |  |
|  |  |  | TGCGCTGTAGACATGATCATTCCCCCGCTCTC | 24 | No hit |  |
|  |  |  | AGCACCTCATCCTGTCCGCCGGCACGCCACCC | 25 | No hit |  |
| CCUG33950 | 53 | Meningitis/CSF | CACCGGGCCCATCCCGGTCGGCCTCCTGAAA | 6 | type I *P. acnes* genomes | TAD locus |
|  |  |  | TCACACCAGTCATCAGCGTCATAGTCCTCTC | 11 | No hit |  |
| CCUG45436 | 55 | Oral cavity | CACCGGGCCCATCCCGGTCGGCCTCCTGAAA | 6 | type I *P. acnes* genomes | TAD locus |
|  |  |  | TCACACCAGTCATCAGCGTCATAGTCCTCTC | 11 | No hit |  |
